# Rapid One-Step Capturing of Native, Cell-Free Synthesized and Membrane-Embedded GLP-1R

**DOI:** 10.3390/ijms24032808

**Published:** 2023-02-01

**Authors:** Lisa Haueis, Marlitt Stech, Eberhard Schneider, Thorsten Lanz, Nicole Hebel, Anne Zemella, Stefan Kubick

**Affiliations:** 1 Fraunhofer Institute for Cell Therapy and Immunology (IZI), Branch Bioanalytics and Bioprocesses (IZI-BB), Am Mühlenberg 13, 14476 Potsdam, Germany; 2 Institute of Biochemistry and Biology, University of Potsdam, Karl-Liebknecht-Str. 24–25, 14476 Potsdam, Germany; 3 3B Pharmaceuticals GmbH, Magnusstraße 11, 12489 Berlin, Germany; 4 Institute of Chemistry and Biochemistry, Freie Universität Berlin, Thielallee 63, 14195 Berlin, Germany; 5 Faculty of Health Sciences, Joint Faculty of the Brandenburg University of Technology Cottbus–Senftenberg, the Brandenburg Medical School Theodor Fontane and the University of Potsdam, 14476 Potsdam, Germany

**Keywords:** cell-free protein synthesis, CFPS, cell-free expression, *Sf*21 cell lysate, G protein-coupled receptors, GLP-1R, membrane, immobilization to magnetic beads

## Abstract

G protein-coupled receptors (GPCRs) are of outstanding pharmacological interest as they are abundant in cell membranes where they perform diverse functions that are closely related to the vitality of cells. The analysis of GPCRs in natural membranes is laborious, as established methods are almost exclusively cell culture-based and only a few methods for immobilization in a natural membrane outside the cell are known. Within this study, we present a one-step, fast and robust immobilization strategy of the GPCR glucagon-like peptide 1 receptor (GLP-1R). GLP-1R was synthesized in eukaryotic lysates harboring endogenous endoplasmic reticulum-derived microsomes enabling the embedment of GLP-1R in a natural membrane. Interestingly, we found that these microsomes spontaneously adsorbed to magnetic Neutravidin beads thus providing immobilized membrane protein preparations which required no additional manipulation of the target receptor or its supporting membrane. The accessibility of the extracellular domain of membrane-embedded and bead-immobilized GLP-1R was demonstrated by bead-based enzyme-linked immunosorbent assay (ELISA) using GLP-1R-specific monoclonal antibodies. In addition, ligand binding of immobilized GLP-1R was verified in a radioligand binding assay. In summary, we present an easy and straightforward synthesis and immobilization methodology of an active GPCR which can be beneficial for studying membrane proteins in general.

## 1. Introduction

G protein-coupled receptors (GPCRs) belong to one of the largest and functionally diverse protein superfamilies. Structurally, they are characterized by seven transmembrane helices, an intracellular C-terminus and an extracellular N-terminus. Their fundamental mode of action is to activate intracellular heterotrimeric G proteins due to conformational change upon stimuli to their extracellular domain. Thus, these receptors are an important link between exogenous stimuli that affect cells and their intracellular signaling [1,2,3]. As GPCRs regulate vital signal transduction pathways, they are of outstanding interest as pharmacological targets. Around 34% of all drugs approved by the US Food and Drug Administration (FDA) act on GPCRs [4]. Keeping in mind that the family of GPCRs includes around 800 members in humans and that there is still a huge number of orphan receptors for whom no ligand has been identified, it is obvious that there is a strong demand for extensive research on this class of membrane proteins [4]. However, the isolation and production of defined pharmaceutically relevant GPCRs, in particular for those where the overexpression results in signaling cascades with cytotoxic effects, in sufficient amounts for functional and structural analysis remains challenging. Although most eukaryotic membrane proteins are found in many tissues, the isolation of sufficient material in adequate concentrations often ends up in tedious and time-consuming lab work. A classical method to produce and subsequently characterize membrane proteins in sufficient quantities is their recombinant overexpression in cell-based systems (in vivo protein production) [5,6,7]. Especially, the overexpression of eukaryotic membrane proteins is often difficult and is mostly accompanied by poor expression levels of functional protein due to high rates of degradation, formation of inclusion bodies and toxicity against the host cells [8,9,10]. Complementary to cell-based approaches, cell-free protein synthesis (CFPS) based on eukaryotic lysates offers a convenient alternative in tackling these difficulties [11,12,13]. For membrane proteins, particularly for those with transmembrane domains, it is mandatory to provide an appropriate membrane for the insertion of the functional protein thus preserving its native folding and function [14,15,16]. Eukaryotic lysates containing endogenous microsomes derived from the endoplasmatic reticulum (ER) of the cells used for lysate preparation, perfectly meet this requirement: due to the natural origin of microsomes, functionally active membrane proteins with their related posttranslational modifications (PTMs), can be co-translationally incorporated into the microsomal membrane to maintain their correct folding and function [12,17,18,19,20,21].

In this study, we aimed to establish an efficient and fast immobilization strategy for GPCRs that maintains their functional and structural integrity using the Glucagon-like peptide-1 receptor (GLP-1R) as a model. GLP-1R belongs to the GPCR family B1 and is of high physiological and pharmacological relevance. The natural ligand of GLP-1R is the peptide hormone GLP-1 which promotes insulin secretion in response to food uptake [22]. GLP-1R is located in multiple tissues in the human body such as pancreatic islets, heart, intestine, kidney and brain and represents an established target for the treatment of type 2 diabetes mellitus [23]. Until now, there are six synthetic GLP-1R agonists approved as pharmaceuticals to treat diabetes mellitus type 2, which have been derived from either GLP-1 itself or its paralogue exendin-4 [24]. Exendin-4 is a naturally occurring 39-amino acid-peptide, which is secreted by the salivary glands of the desert Gila monster (*Heloderma suspectum*). It is known to be a potent agonist of mammalian GLP-1R, which shares 53% sequence homology with human GLP-1, but is resistant to enzymatic degradation by dipeptidyl peptidase-4 (DPP-4) [25]. It was also shown that both exendin-4 and GLP-1 activate GLP-1R with similar potency, whereas the N-terminally truncated form of exendin-4 (9–39), is a potent antagonist of GLP-1R [26]. While GLP-1R agonists are well established for the treatment of hyperglycemia in the context of diabetes, GLP-1R antagonists are nowadays in the focus to fight hypoglycemia [27].

Several methods and platforms which enable the discovery and characterization of new leads for drug discovery (e.g., mRNA display, phage display, surface plasmon resonance) require the immobilization of target proteins onto solid scaffolds. The immobilization of GPCRs in their native and pharmacologically relevant conformation requires consideration of their complex molecular structure and folding which strongly relies on the hydrophobic matrix surrounding the protein. In order to meet these requirements, complex methods have been developed in the past (reviewed in [28]). In general, there are two basic experimental strategies to make GPCRs available as immobilized targets: either by adaptation of the GPCR itself or by adaptation of the GPCR surrounding matrix. The first strategy requires the modification of the membrane protein, e.g., by introducing epitope tags for purification [29,30] and/or by designing GPCR mutants, which are structurally more stable compared to their wild type [31]. Usually, affinity tags can be located exclusively at the N-terminal or C-terminal end of the protein which bears the danger of an impaired GPCR functionality. Apart from that, the design of GPCR mutants which are more stable is laborious and needs to be adapted individually for each membrane protein target of interest. The adaptation of the GPCR surrounding matrix usually requires solubilization and reconstitution of the target protein to an artificial lipid environment (e.g., by incorporation into proteoliposomes or nanodiscs) [15,32] which may alter receptor structure and function and again needs to be optimized for each individual receptor. Approaches for the immobilization of GPCRs directly from crude membranes without prior purification or solubilization exist but are mainly based on overexpression of GPCRs in eukaryotic cell cultures [29,30,33]. Thus, improved techniques which allow a convenient and fast synthesis and immobilization of difficult-to-express membrane proteins are urgently needed.

Here we compare two eukaryotic microsome-containing cell-free systems (*Sf*21 and CHO) in order to establish a fast synthesis and immobilization pipeline for GLP-1R. Initially, we planned to achieve this goal by using the amber suppression technology to introduce a Biotin–lysine into GLP-1R at a defined amino acid position. Surprisingly, we found that microsomal membrane-embedded GLP-1R spontaneously immobilizes to the surface of magnetic Neutravidin beads with high yield and in a stable way, independent of the presence of an introduced Biotin moiety. The extracellular domain (ECD) of the GPCR was shown to be accessible, as an ECD-specific monoclonal antibody could bind to GLP-1R. Moreover, we were able to demonstrate the binding of a conformation-specific antibody which can block the GLP-1 binding site of the ECD directly and thereby acts as a competitive antagonist of native GLP-1. Finally, membrane protein preparations of cell-free synthesized and bead-immobilized GLP-1R were shown to bind specifically to a radioactively labeled exendin-4 peptide ligand.

## 2. Results

### 2.1. Cell-Free Synthesis of Glycosylated GLP-1R in Eukaryotic Lysates

**Figure 1:**
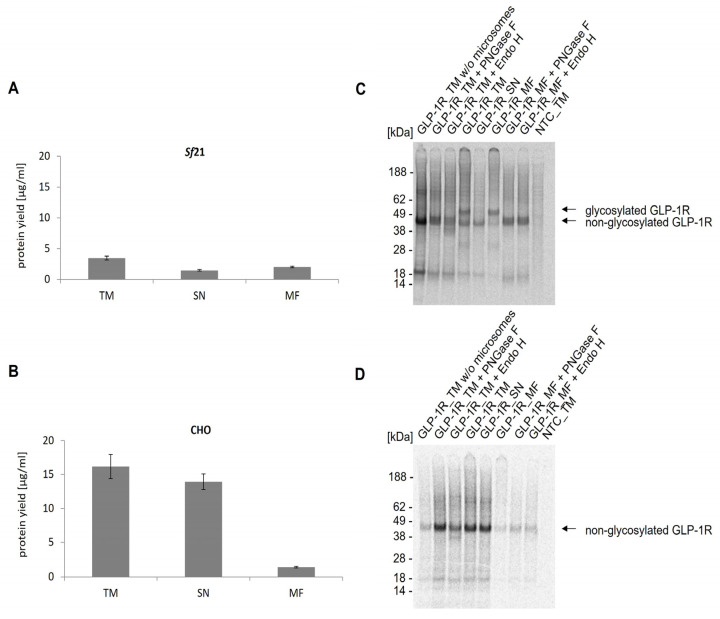
Analysis of cell-free synthesized GLP-1R in *Sf*21 and CHO lysate. Cell-free synthesized GLP-1R was analyzed in three lysate fractions (translation mixture (TM), soluble fraction (SN), microsomal fraction (MF)) and in TM of a cell-free synthesis reaction performed with microsome depleted lysate using the *Sf*21 (**A**) and the CHO cell-free system (**B**). Protein yields of 14C-leucine labeled GLP-1R were determined by precipitating aliquots of TM, SN and MF in hot TCA followed by liquid scintillation measurement using a scintillation counter (Hidex 600 SL). Standard deviations were calculated from triplicates. (**C**) Autoradiogram of GLP-1R synthesized using the cell-free *Sf*21 system. The qualitative analysis shows the expected molecular weight of the synthesized protein in its glycosylated and non-glycosylated form. Translation mixture (TM) of cell-free synthesized GLP-1R was separated by centrifugation into SN and MF. In order to prove the glycosylation, the TM and MF were treated with the enzymes PNGase F and Endo H. (**D**) Autoradiogram of GLP-1R synthesized in cell-free CHO system. No template control (NTC) was carried along as negative control.

As already shown, translationally active lysate based on *Spodoptera frugiperda* 21 (*Sf*21) and Chinese hamster ovary (CHO) cells can be generated, harboring endogenous membrane vesicles having their origin in the ER and thereby allowing for co-translational translocation and incorporation of de novo synthesized proteins into the microsomal membrane. This is a crucial prerequisite for the implementation of posttranslational modifications [34,35,36,37]. To assess which system was most suitable for the synthesis of GLP-1R, cell-free synthesis was performed in coupled batch reactions based on either *Sf*21 or CHO lysate and compared with regard to protein yield and glycosylation. The efficiency of CFPS was monitored after 3 h of incubation at 27 °C (*Sf*21) or 30 °C (CHO). The trichloracetic acid (TCA) precipitation data of the translation mixture (TM) showed that the protein yields in CHO (16 µg/mL) were notably higher than in *Sf*21 (4 µg/mL) (Figure 1A,B). By analyzing the soluble fraction (SN) of TM and the microsomal fraction (MF), a different translocation efficiency between both lysates was observed. In contrast to CHO lysate, where approximately 9% of the target protein was detected in the MF, the translocation efficiency in *Sf*21 lysate was much higher (around 59%).

The molecular weight and protein integrity of cell-free synthesized and 14C-labeled GLP-1R was characterized by sodium dodecyl sulfate polyacrylamide gel electrophoresis (SDS-PAGE) followed by autoradiography. Distinct protein bands showed the successful cell-free synthesis of GLP-1R using *Sf*21 and CHO lysates (Figure 1C,D). The autoradiogram of GLP-1R synthesized in *Sf*21 lysate (Figure 1C) revealed two protein bands, migrating at around 45 kDa and 50 kDa. As the upper protein band appeared only in the TM and MF, but not in SN where no microsomes were expected, it was assumed that the molecular mass of GLP-1R was increased due to N-glycosylation. Natural GLP-1R is known to be N-glycosylated at positions 63, 82 and 115 [38]. In order to prove the assumption of GLP-1R N-glycosylation within the cell-free system, the protein was treated with Peptide N-glycosidase F (PNGase F), an enzyme which specifically cleaves N-linked oligosaccharides from glycoproteins. For further characterization of the glycosylation the target protein was also treated with endoglycosidase H (Endo H), which removes only N-linked high mannose carbohydrates but not highly processed complex structures from glycoproteins [39]. Referring to the autoradiogram (Figure 1C), it could be clearly seen that the bands with higher molecular weight disappeared after PNGase F and Endo H treatment. To further evaluate the ability of ER-microsomes for glycosylation, cell-free protein synthesis was additionally performed in microsome-depleted lysate. Here, only the band with lower molecular weight could be seen, which proved that the microsomes were necessary to achieve glycosylated GLP-1R. While cell-free synthesis in CHO lysates resulted in higher GLP-1R yields compared to the *Sf*21 system, only the *Sf*21 system allowed for the synthesis of the glycosylated GPCR. Since glycosylations are known to be important for the functionality of GLP-1R [40,41], the *Sf*21 system was favored over the CHO system for all subsequent experiments.

### 2.2. Site-Specific and Co-Translational Biotinylation of a GLP-1R Amber Mutant Using Precharged tRNAs

**Figure 2:**
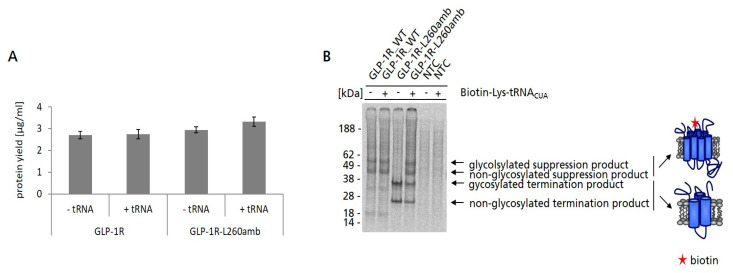
Site-specific biotinylation of GLP-1R-L260amb using Biotin-Lys-tRNA_CUA_. (**A**) For the determination of GLP-1R and GLP-1R-L260amb protein yields, 5 µL of TM was precipitated by hot TCA precipitation followed by liquid scintillation counting. Standard deviations were calculated from triplicates. (**B**) For qualitative characterization by autoradiography, a 5 µL aliquot of the translation reaction mixture (TM) was precipitated with acetone. The resulting pellets were resolved in sample buffer. Samples were then electrophoretically separated on a 10% SDS-PAGE gel followed by autoradiography. No template control (NTC) was carried along as negative control. Full-length suppression product and termination product are indicated by arrows. The structural model of the seven transmembrane domains of GLP-1R is indicated on the right. The red star represents the incorporated Biotin-label.

Our initial strategy to immobilize cell-free synthesized GLP-1R was based on the amber suppression technology to incorporate a Biotin moiety at a defined position within the GLP-1R sequence using a pre-charged tRNA (Biotin-Lys-tRNA_CUA_). To achieve this goal, an amber mutant of GLP-1R was generated (GLP-1R-L260amb). For this, the amber stop codon was introduced to the second intracellular loop replacing leucine at position 260 (L260) as it was shown that a mutation at this position had no negative effect on the function of GLP-1R [42]. Cell-free synthesis of GLP-1R-L260amb was performed in the batch-based *Sf*21 system in presence and absence of Biotin-Lys-tRNA_CUA_. As a control, also wildtype GLP-1R was synthesized under the same conditions. Based on TCA precipitation results, it was observed that the total protein yield of wildtype GLP-1R (approx. 2.7 µg/mL) and its amber mutant (approx. 3 µg/mL) were not influenced by the presence of Biotin-Lys-tRNA_CUA_ (Figure 2A). As could be seen in the autoradiogram, supplementation of GLP-1R-L260amb cell-free reaction with Biotin-Lys-tRNA_CUA_ resulted in four distinct protein bands, corresponding from top to bottom to glycosylated suppression product, non-glycosylated suppression product (58 kDa), as well as glycosylated and non-glycosylated termination product (30 kDa). As expected, the synthesis of GLP-1R-L260amb in absence of Biotin-Lys-tRNA_CUA_ resulted in the detection of the glycosylated and non-glycosylated termination product only (Figure 2B). While the presence of suppression product indicated the successful incorporation of Biotin–lysine into cell-free synthesized GLP-1R-L260amb, the strong appearance of termination product suggested a limitation of the efficiency to incorporate the non-canonical amino acid. In addition, “read-through” events, where a natural amino acid is incorporated at the position of the amber stop codon, can lead to full-length suppression products which do not carry the desired Biotin label. However, if such “read-through” products were synthesized, their concentration was too low to be detectable by autoradiography. Nevertheless, it was expected that only (full-length) biotinylated species of GLP-1R should be captured on magnetic Streptavidin/Neutravidin beads. Thus, GLP-1R-L260amb synthesized in the presence of Biotin-Lys-tRNA_CUA_ was subsequently subjected to bead-based purification using magnetic Streptavidin/Neutravidin beads.

### 2.3. Immobilization of Biotinylated GLP-1R-L260amb onto Magnetic Beads

**Figure 3:**
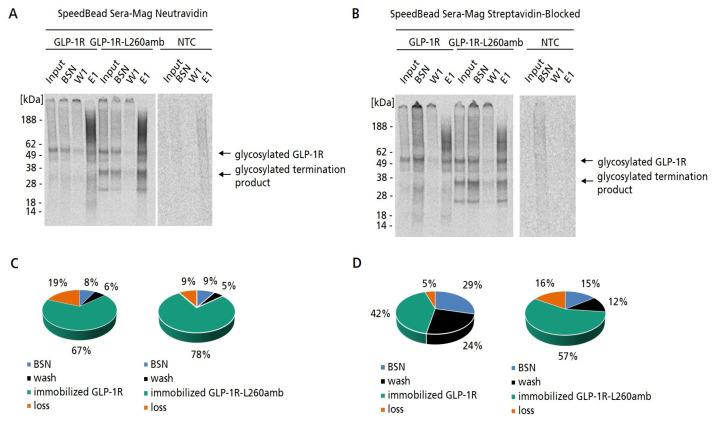
Purification of cell-free synthesized, membrane-embedded GLP-1R and GLP-1R-L260amb comparing two different types of magnetic beads. Synthesis of GLP-1R was performed using the *Sf*21 cell-free system in batch mode. For the synthesis for GLP-1R-L260amb Biotin-Lys-tRNA_CUA_ was supplemented to enable the synthesis of full-length suppression product. (**A**,**B**) Autoradiograms showing the different purification fractions of microsomal membrane-embedded GLP-1R and GLP-1R-L260amb purified using either SpeedBead Sera-Mag Neutravidin beads or SpeedBead Sera-Mag Streptavidin-blocked magnetic particles: For acetone precipitation and further analysis by SDS-PAGE 4% from the microsomal fraction (MF) before purification (input) and 70% of binding supernatant (BSN), washing fraction 1 (W1) and elution fraction 1 (E1) were obtained. No template control (NTC) was carried along as negative control. (**C**,**D**) Pie chart showing the percentage distribution of the fractions collected during the purification of GLP-1R and GLP-1R-L260amb using either SpeedBead Sera-Mag Neutravidin beads (**C**) or SpeedBead Sera-Mag Streptavidin-blocked magnetic particles (**D**). Amount of cell-free synthesized protein which was added to the beads as input, was set to 100%.

In order to prove the successful biotinylation of GLP-1R, MF preparations of GPLP-1R-L260amb synthesized in the presence of Biotin-Lys-tRNA_CUA_ were subjected to a bead-based purification strategy where we compared the performance of two different types of magnetic beads, SpeedBead Sera-Mag Streptavidin-blocked and SpeedBead Sera-Mag Neutravidin magnetic particles (GE). We decided on these two types of magnetic beads as they are well known and well validated. Besides the classical Neutravidin magnetic particles we chose the Streptavidin-blocked particles as we wanted to minimize unspecific binding between the microsomes of the eukaryotic cell-free system and the magnetic beads. The MF was chosen since it was the aim to purify exclusively microsomal membrane-embedded GLP-1R-L260amb, but not the non-translocated protein species. Wildtype GLP-1R was synthesized in parallel as a biotin-free negative control and subjected to the same purification protocol. As cell-free synthesized proteins were 14C-labeled, the purification fractions (binding supernatant, washing fraction and elution fraction) were qualitatively and quantitatively analyzed by autoradiography (Figure 3A,B) and scintillation measurement. Based on the autoradiograms, it was observed that for both types of magnetic beads, distinct bands of GLP-1R-L260amb were detectable in the elution fraction. Surprisingly, these results were also true for the wildtype construct. As there was no incorporated Biotin label expected in wildtype GLP-1R; the capturing of this membrane protein preparation on Neutravidin or Streptavidin beads was not expected. However, both GLP-1R and GLP-1R-L260amb were captured with a comparable efficiency on both types of magnetic beads. This result indicated that the immobilization of GLP-1R-L260amb proceeded independently of the site-specifically introduced Biotin. Comparing the immobilization efficiencies of SpeedBead Sera-Mag Streptavidin-blocked and SpeedBead Sera-Mag Neutravidin magnetic particles, it was observed that the efficiency using Neutravidin beads was much higher (67–78%) compared to Streptavidin beads (42–57%) (Figure 3C,D). Interestingly, the Coomassie-stained SDS-PAGE gels showing the different purification fractions revealed a strong background of proteins with varying molecular weights in the elution fraction (Figure S1). The background proteins might result from endogenous, naturally biotinylated MF proteins that may have been captured on the Neutravidin/Streptavidin magnetic beads. To further investigate this question, the capturing of MF-embedded GLP-1R was analyzed after pre-treatment of beads with soluble Biotin. If the binding of MF-embedded GLP-1R was mediated by naturally occurring biotinylated MF proteins, a reduction or even a complete inhibition of the binding was expected after blocking the beads with soluble Biotin. Interestingly, the autoradiogram revealed no difference in GLP-1R band intensity using blocked or unblocked Neutravidin beads (Figure S2), indicating that GLP-1R and endogenous lysate proteins were captured independently of Biotin. In contrast, the binding of a biotinylated control protein was almost completely inhibited using the Biotin-blocked Neutravidin beads, while a clear band of the protein was detectable in the elution fraction when the beads were unblocked (Figure S3). From this we concluded that GLP-1R and endogenous lysate proteins were captured independently of Biotin, possibly by hydrophobic interactions of the microsomal membranes with the bead surface.

### 2.4. Analysis of Microsomal Membrane-Embedded and Bead-Captured GLP-1R by Confocal Laser Scanning Microscopy (CLSM)

**Figure 4:**
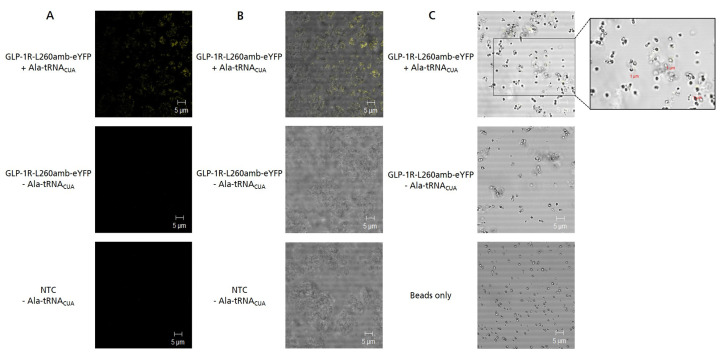
Fluorescence analysis of GLP-1R-L260amb-eYFP fusion protein. GLP-1R-L260amb-eYFP was synthesized in the *Sf*21 cell-free system in batch mode either in presence of Ala-tRNA_CUA_ to obtain fluorescent suppression product or in absence of Ala-tRNA_CUA_ to obtain non-fluorescent termination product. For the detection of background fluorescence, no template control (NTC) was analyzed either. (**A**) Fluorescence image of prepared samples. After cell-free synthesis, an aliquot of 20 µL translation mixture (TM) was transferred to an IBIDI slide and analyzed by confocal laser scanning microscopy. Excitation wavelength: 488 nm (eYFP) and 633 nm (bright field), 60× objective with oil, 2.3× zoom. (**B**) Overlay of confocal fluorescence image and brightfield to visualize colocalization of microsomes and fluorescence signal. (**C**) Overlay of confocal fluorescence image and brightfield of magnetic beads incubated with microsomal membrane-embedded GLP-1R-L260amb-eYFP. In detail, the microsomal fraction (MF) of GLP-1R-L260amb-eYFP was incubated with 10 µg SpeedBead Sera-Mag Neutravidin beads for 1 h at 4 °C, washed twice and transferred to an IBIDI-slide for fluorescence analysis. As a control, untreated beads were analyzed in parallel. For better visibility, the contrast of all images was adjusted equally.

CLSM was applied to analyze the localization of cell-free synthesized GLP-1R within the lysate and to further characterize the interaction between microsomes and magnetic beads. In order to visualize the membrane embedment of GLP-1R, a GLP-1R-L260amb- enhanced yellow fluorescent protein (eYFP) fusion protein was generated. As expected, fluorescence signals could only be observed when GLP-1R-L260amb-eYFP was synthesized in the presence of a pre-charged tRNA (Ala-tRNA_CUA_), which addressed the internal amber stop codon and enabled the synthesis of full-length suppression product (Figure 4A). In the overlay pictures (Figure 4B), a clear co-localization of GLP-1R-L260amb-eYFP fluorescence and microsomal structures could be seen, indicating that the membrane protein was embedded in the lipid bilayer as expected. To study the interaction between the magnetic beads and MF-embedded GLP-1R, SpeedBead Sera-Mag Neutravidin beads were incubated with MF-embedded GLP-1R-L260amb-eYFP for 1 h at 4 °C and subsequently analyzed using CLSM. Interestingly, we observed that the beforehand monodisperse bead solution became clumpy and formed aggregates when incubated with the MF-embedded GLP-1R-L260amb-eYFP (Figure 4C). In addition, we noticed that the microsomal membranes seemed to form an irregular layer around single beads but also bead agglomerates. Moreover, even microsome agglomerates seemed to stick to the magnetic beads. This interaction was found to be strong enough to endure repeated bead separation, washing and processing.

### 2.5. Analyzing the Accessibility of Bead-Captured GLP-1R ECD for Antibody Binding

**Figure 5:**
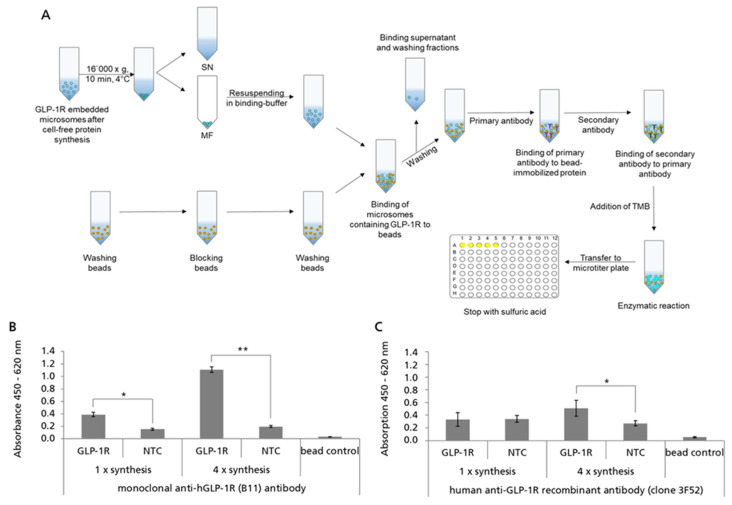
Probing the accessibility of bead-captured GLP-1R extracellular domain (ECD) by bead-based ELISA. GLP-1R was synthesized using the batch-formatted *Sf*21 cell-free synthesis system (1×) as well as repetitive cell-free protein synthesis (4×). Translation mixture (TM) was fractionated in supernatant (SN) and microsomal fraction (MF). (**A**) Scheme of the implementation of the bead-based ELISA with cell-free synthesized GLP-1R. (**B**,**C**) MF was immobilized using magnetic SpeedBead Sera-Mag Neutravidin beads without elution of target protein to obtain bead-captured GLP-1R for further analysis. Monoclonal anti-hGLP-1R antibody (B11, Santa Cruz Biotechnology) and human anti-GLP-1R recombinant antibody (clone 3F52 creative biolabs) were analyzed for the binding of microsomal membrane-embedded and bead-captured GLP-1R ECD. No template control (NTC) and a bead control were carried along as negative controls. Standard deviations were calculated from duplicates (antibody B11) or quadruplicates (antibody 3F52). For the statistical analysis, a *t*-test for two independent samples was performed with *p* ≤ 0.05 (*) and *p* ≤ 0.01 (**).

In order to evaluate the orientation and accessibility of GLP-1R in microsome-embedded and bead-captured preparations, we analyzed their binding reactivity with ECD-specific monoclonal antibodies (Figure 5A). For this, GLP-1R was synthesized in cell-free reactions based on *Sf*21 cell lysate and the MF was harvested by centrifugation and captured on SpeedBead Sera-Mag Neutravidin beads. Subsequently, bead preparations were incubated with anti-hGLP-1R antibody B11 which is reported to bind to the ECD (amino acids 91–145) of GLP-1R [43]. The detection of bound antibody was enabled using a secondary anti-mouse antibody conjugated with HRP. An increased absorbance signal was detected for bead preparations coated with microsomal membranes harboring GLP-1R compared to the controls (NTC and untreated beads), demonstrating that the ECD of GLP-1R was accessible for the binding of the monoclonal antibody (Figure 5B). Moreover, the signal for GLP-1R was tremendously increased when using bead preparations coated with microsomal membranes harboring an enriched proportion of GLP-1R. Enrichment of GLP-1R was achieved by performing four repetitive cell-free protein synthesis reactions using the same batch of microsomes. In addition to antibody B11, anti-GLP-1R antibody 3F52 was analyzed for binding to standard and GLP-1R enriched bead preparations. Unlike antibody B11, 3F52 is reported to bind to the GLP-1 binding site of the ECD, thereby acting as a competitive antagonist of native GLP-1 [44,45]. In the bead-based ELISA, a clear difference between the GLP-1R and NTC signal was only observed when using the GLP-1R enriched bead preparations (Figure 5C). In order to monitor unspecific background binding, magnetic beads were also incubated with an unrelated GPCR (GIPR) and analyzed in parallel. For both, anti-GLP-1R antibody signals obtained for the unrelated GPCR (GIPR) were comparable to NTC signals demonstrating that the binding was not influenced by the presence of a cell-free synthesized GPCR or its encoding DNA or mRNA. Based on these results, we assumed that besides monoclonal antibodies, peptide ligands should also have access to the ECD of GLP-1R.

### 2.6. Binding of Exendin-4 Peptide Ligand to Bead-Captured GLP-1R Preparations

**Figure 6:**
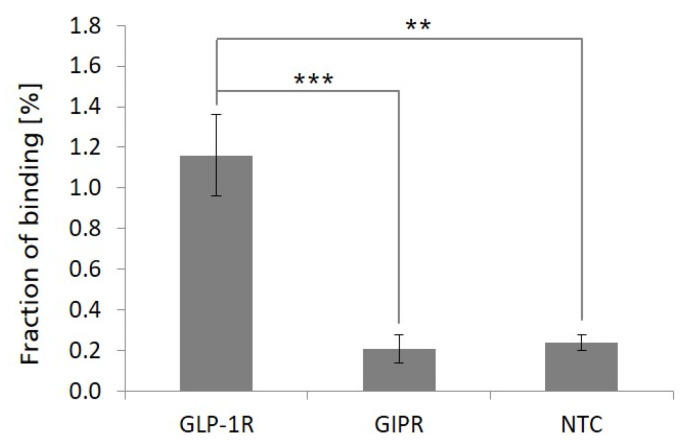
Ligand-binding assay of radioactive-labeled exendin-4 peptide ligand to bead-captured, microsome membrane-embedded GLP-1R. Microsomal fractions of cell-free synthesized GLP-1R were harvested from a standard *Sf*21 in vitro transcription/translation reaction and incubated with SpeedBead Sera-Mag Neutravidin beads. In addition, microsomal fractions of cell-free synthesized GIPR and a no template control (NTC) reaction were treated likewise and used as controls to monitor background binding activities. Bead-captured microsomal fractions were analyzed for the binding of radioactive-labeled exendin-4 peptide ligand. Percentage values show the fraction of radioactive-labeled peptide ligand bound to bead-captured microsomal fractions. GLP-1R and NTC values were calculated from quadruplicates, GIPR values were calculated from triplicates. For the statistical analysis, a *t*-test for two independent samples was performed with *p* ≤ 0.01 (**) and *p* ≤ 0.001 (***).

To analyze the ligand-binding properties of bead-captured, cell-free-synthesized, microsome membrane-embedded GLP-1R, a radio ligand-binding assay was performed. For this, bead-captured GLP-1R MF preparations were prepared as described above and analyzed for the binding of a radioactive-labeled exendin-4 peptide ligand. Using this approach, we were able to detect significantly increased binding signals on GLP-1R MF bead preparations compared to GIPR MF or NTC MF bead preparations (Figure 6). The addition of an excess of synthetically produced, unlabeled exendin-4 peptide (100-fold molar excess in relation to radioactive-labeled exendin-4 peptide ligand) efficiently inhibited the GLP-1R-specific elevated binding signals on GLP-1R bead preparations nearly completely (>90% inhibition), demonstrating the specificity of the observed GLP-1R-related binding signal.

## 3. Discussion

In view of the medical need for properly folded membrane protein structures with simultaneous efficient integration into a lipid bilayer and posttranslational modifications, cell-free protein synthesis based on eukaryotic lysates has been shown to provide a versatile platform [11,19,46,47,48,49]. In this study, we demonstrate the cell-free synthesis of GLP-1R in microsome-containing systems derived from cultured *Sf*21 and CHO cells (Figure 1). It has been shown before that *Sf*21 and CHO cell-free systems allow for the synthesis of post-translationally modified proteins due to the presence of endogenous microsomes derived from the ER [37,50,51]. GLP-1R is known to be N-glycosylated within the ER at three positions (Asn63, Asn82 and Asn115) [38,40]. Consistently with GLP-1R expressed in CCL39 fibroblasts [52], HEK293 [53,54] and CHO cells [38,52,53,54], cell-free synthesized GLP-1R was detected as a mixture of non-glycosylated and glycosylated receptor (Figure 1C). Since microsomal structures of the lysates were ER derived, only high-mannose sugar moieties were expected, as more complex carbohydrates are processed in the Golgi of eukaryotic cells [37]. As EndoH exclusively removes N-linked high mannose carbohydrates but not highly processed complex structures from glycoproteins [39], our data indicate that the receptor underwent N-linked glycosylation within the *Sf*21 cell-free system. Interestingly, no glycosylation was detected when using the CHO cell-free system, although N-linked glycosylation of GLP-1R has been shown in CHO cells [38,40] indicating that the conditions within the CHO cell-free system, possibly the concentration of signal peptide recognition particles as well as active translocon complexes within ER microsomes, were not appropriate to enable GLP-1R glycosylation. The influence of N-glycosylation with regard to GLP-1R cell surface expression and function has been extensively studied using eukaryotic cell cultures. Collectively, it was shown that N-linked glycosylation of GLP-1R is important for the trafficking, maturation and activity of the receptor [38,40,52,54]. Therefore, we decided to continue our studies with a GLP-1R as native as possible and preferred the *Sf*21 over the CHO system, despite the higher yields of the latter system.

There are many strategies to immobilize membrane proteins for further characterizations and applications [28]. Especially, the immobilization to magnetic beads is of enormous interest for various display technologies and ligand-binding studies [55,56,57,58]. In our study, we initially wanted to realize the immobilization of cell-free synthesized GLP-1R to magnetic beads by site-specifically introducing a Biotin moiety to the GPCR (Figure 2). Interestingly, we found that the microsomal membranes harboring GLP-1R adsorbed spontaneously and independently of the introduced Biotin to the surface of magnetic beads (Figure 3 and Figure 4). However, it was obvious that the target protein was not pure, as there were also endogenous microsomal proteins immobilized to the beads. The direct adsorption of crude membranes on the hydrophobic surface of plastic beads has been shown before for the acetylcholine receptor located in membranes of the electric organ of *Torpedo californica* [59]. In view of this publication and the Biotin-blocking experiment (Figure S3), it seemed likely that hydrophobic interactions of microsomal membranes with the bead surface were facilitating the immobilization. Looking deeper at the chemistry of the beads, electrostatic interactions between beads and microsomes could also be considered. The core of the Neutravidin beads was a carboxyl bead to which Neutravidin was coupled via carbodiimide chemistry, whereby some unreacted carboxyl groups and a very small amount of sulphonate groups were expected (manufacturer’s information). Instead, Streptavidin beads consisted of a polystyrene core encapsulated in two layers of magnetite covalently bound to Streptavidin [60] and further encapsulated with proprietary polymers such as polyamines and polycarboxylic acids (manufacturer’s information). The bead chemistries seem to have an impact on purification efficiency, as we observed different purification efficiencies for the two bead types (Figure 3C,D). As we assumed the presence of ribosomal RNA and mRNA at the surface of the microsomes giving them a rather negative charge, we suspected that a positively charged bead surface, possibly mediated by polyamines, would also contribute to the binding. Interestingly, the interaction between microsomes and beads was found to be stable enough to endure repeated washing and magnetic separation steps, even in the presence of increasing concentrations of detergents. The observation of a non-covalent, but very stable interaction of microsomes and bead surface opened up the possibility to use this kind of bead preparation directly to study the functionality of GLP-1R, while neglecting the need for a site-specific modification of the GPCR as originally planned for this purpose.

For performing ligand-binding experiments, it was crucial that the ECD of GLP-1R was accessible. During cell-free synthesis the target protein gets incorporated into the microsomal membrane due to signal peptide-induced translocation [12,46,50]. In our study, the natural signal sequence of GLP-1R was replaced by the signal sequence of honey bee melittin which has been shown to allow for efficient translocation in eukaryotic cell-free systems [21]. In theory, the ECD of microsomal membrane-embedded GLP-1R should face the microsomal lumen. Therefore, we were seeking to find experimental conditions under which peptide GLP-1R ligands, such as exendin-4, could reach their binding site within the ECD. In previous studies focusing on the cell-free synthesis and functional analysis of the endothelin B (ET-B) receptor, it was shown that the binding of the natural ligand endothelin-1 (ET-1) was possible upon perforation of ER-derived microsomes using 0.03% Brij35 [12]. We considered that the detergent Tween, which was by default present in blocking, washing and binding buffers during magnetic bead processing, would perforate the microsomes as well so that their lumen should become accessible for ligands. In order to prove this assumption, we decided to analyze GLP-1R bead preparations for the binding of antibodies specific for the ECD of GLP-1R. If the ECD of bead immobilized GLP-1R was accessible, these antibodies should be able to bind. This was exactly what we detected for the monoclonal anti-hGLP-1R antibody B11 (Santa-Cruz) as well as anti-GLP-1R antibody 3F52. In addition, an inverted orientation of bead-immobilized microsomes (“inside-out microsomes”), possibly as a cause of the detergent treatment [61] and/or contact to the beads, could be another explanation for the accessibility of the microsomal lumen for GLP-1R ECD specific antibodies.

As a member of the GPCR class B family, GLP-1R contains a ~15 kDa N-terminal ECD which is essential for binding to the C-terminal part of the cognate peptide hormones. In particular, the α -helix of the ECD is known to be important for ligand binding and specificity of GLP-1R [62,63]. In the context of this study, it is important to note that the GLP-1R epitope of antibody 3F52 almost exclusively involves this α -helix (Leu32-Arg43). By directly binding to the α-helix, antibody 3F52 has been reported to block the GLP-1 binding site, thereby acting as a competitive antagonist of native GLP-1 [45]. In this context Hennen et al. suggested a tilted conformation of 3F52 antibody-bound GLP-1R ECD. More recently, the crystal structure of full-length GLP-1R in an inactive state was reported revealing that a unique closed conformation of the ECD is favored in the absence of orthosteric ligands such as GLP-1 [64]. Further, this study suggested that a large degree of conformational dynamics within the GLP-1R ECD is necessary for binding of GLP-1. In view of these findings, our data (Figure 5C) indicate that the ECD of cell-free synthesized and immobilized GLP-1R was presented in a conformation appropriate for the binding of the ligand-blocking antibody 3F52, possibly as a consequence of conformational dynamics within the GLP-1R ECD. Compared to antibody B11, 3F52 showed a significantly lower signal-to-noise ratio, which might be explained by different binding sites of the two monoclonal antibodies indicating that only a fraction of membrane-embedded and bead-immobilized GLP-1R was presented with a native conformation. The assumption of a structural and functional integrity of cell-free synthesized GLP-1R ECD was further strengthened by data demonstrating the specific binding of 35S-methionine-labeled exendin-4 peptide ligand (Figure 6). Though the determination of ligand-binding dissociation constants was not within the scope of this study, it is noteworthy that the binding affinity of another cell-free synthesized GPCR, ET-B receptor and its ligand ET-1 was found to be in good correlation with the dissociation constant determined in vivo in human tissues (28.6 pM for cell-free ET-B receptor compared to 10 to 150 pM in vivo) [12]. The *Sf*21 cell-free system used in our study was comparable to the one applied by Zemella and coworkers, thus demonstrating the feasibility of the system to produce active GPCRs in terms of ligand binding [12]. However, based on our data, we cannot draw conclusions on the capability of cell-free synthesized GLP-1R to activate G proteins. For this, further studies need to be conducted, for example using approaches based on Förster (FRET) and bioluminescence resonance energy transfer (BRET) [65].

Despite high GPCR production yields using in vitro and in vivo expression systems, the active ligand-binding fraction of GPCRs can be frequently low (<1%) [12,15,66]. One reason for this can be that the lipid composition was not optimal for the heterologously expressed target protein. It is well known that GPCR functionality and signaling are strongly influenced by the lipid composition of the GPCR-embedding membrane [67]. The lipid composition varies between different cell types, but also between cellular membranes within the same cell. [68]. Likewise, the lipid composition of *Sf*21 ER-derived microsomes is expected to be different from the outer cell membrane of human cells (e.g., pancreatic β-cell), where human GLP-1R is naturally expressed (among other cell types and organs) [44], which probably bears an influence on GLP-1R functionality [69,70]. Due to the open nature of cell-free reactions, the lipid composition within the system can be easily adapted. For example, the successful improvement of GPCR activity by varying the lipid composition has been reported for ETB receptor produced in a cell-free *Escherichia coli* system [71]. Referring to the microsome-containing *Sf*21 system, one possibility to boost the fraction of active GLP-1R could be to modify the lipid composition of the ER microsomes, e.g., by supplementation with lipids/sterols most prominently found in the plasma membrane of human cells.

The focus of this study was to establish a convenient methodology to make functional GPCRs available as immobilized target proteins. To our knowledge, this is the first report of a cell-free synthesized GPCR embedded in a natural ER-like membrane immobilized to magnetic beads. Techniques to immobilize membrane proteins to various surfaces are highly in demand in order to functionally characterize these proteins and to screen for new lead candidates in drug discovery. The modification of the membrane protein itself by fusing it to affinity tags is a common strategy for the subsequent immobilization. For example, Ott and coworkers demonstrated the immobilization of a detergent-solubilized functional ĸ-opioid receptor which has been extracted from lipid bilayers of HEK293T cells. Immobilization of the receptor was achieved by fusing it to a C-terminal myc tag, making the GPCR amenable for binding to magnetic beads coated with Protein G and anti-myc antibody [29]. In a more recent study, haloalkane dehalogenase (Halo) was used as an immobilization tag fused to the ß2-adrenoceptor (ß2-AR), angiotensin II type 1 (AT1) and angiotensin II type 2 (AT2) receptors [30]. However, attachment strategies of membrane proteins via epitope tags (e.g., histidine tag, Halo tag, myc tag) and introduction of Biotin groups typically require the modification of the GPCR, which may alter the receptor’s structure and ligand-binding parameters. In addition, membrane proteins with many hydrophobic domains, such as GPCRs, require a lipid environment to maintain their native conformation and full functionality [67]. Therefore, researchers have developed strategies to immobilize GPCRs directly from their native membrane environment, reconstituting them in an artificial lipid microenvironment [33,47,72,73,74]. In our approach neither the GPCR itself was modified, nor was the membrane lipid composition changed to achieve immobilization. The main strength of our strategy lies in its simplicity and efficiency. By using cell-free systems containing endogenous microsomes, a natural membrane environment for co-translational translocation and membrane protein embedment is provided. Immobilization of de novo synthesized GPCRs has been realized without any further modification of the target protein, by simply mixing the GPCR-containing microsomes with magnetic Neutravidin beads, thereby circumventing labor-intensive purification steps. Due to the adsorption of the microsomal membranes to the beads, not only de novo synthesized GLP-1R was captured, but also endogenous proteins originating from the ER microsomes (Figure S1). Therefore, appropriate controls were included to the antibody and peptide-binding experiments, representing beads coated with microsomes without GLP-1R (NTC control) or with a cell-free synthesized, unrelated GPCR (GIPR control). For these controls, a background signal was observed higher than the untreated bead control (Figure 5B,C and Figure 6). As insects in general and *Sf*21 cells in particular do not express GLP-1R or other GPCRs with significant similarity (BLAST search based on human GLP-1R amino acid sequence according to UniProtKB—P43220), endogenously expressed GLP-1R was excluded as a reason for the observed background signal. Instead, we rather assumed unspecific interactions of exendin-4 peptide ligand and GLP-1R-specific antibodies with the microsomal membrane (e.g., by electrostatic interactions) and/or associated ribosomes and mRNA [75]. Background signals may also arise from unspecific interactions of binders with translation-associated proteins as well as cytoskeleton proteins such as actin, dynosin and myosin which have been shown to be co-localized with the *Sf*21 microsomes used in this study.

With regard to screening assays, the observed complexity of bead-adsorbed microsomes can be expected to be challenging due to the multitude of offered binding sites and epitopes. However, strategies to suppress non-specific binding are well established for in vitro display technologies (e.g., blocking procedures, preclearings and negative selections) [76] representing crucial strategies when using complex membrane protein preparations as introduced in this study. In the last decade, novel ligand screening technologies based on DNA-encoded chemical libraries (DELs) and affinity selection mass spectrometry (MS) have been specifically adapted to (purified) membrane proteins and even more challenging to non-purified membrane proteins embedded in natural membranes [77]. Recently, Huang et al. reported successful DEL screenings against endogenously expressed membrane proteins on live cells (e.g., Hela cells) [78]. Quin et al. reported a promising affinity selection MS approach enabling ligand screenings towards crude insect cell membranes containing overexpressed GLP-1R transmembrane domain [79]. Here we suggest that the cell-free synthesis of GPCRs can be a valuable tool significantly complementing these methods as target proteins can be produced fast and independent of living cells which bears advantages for low abundant targets and targets harming cell vitality. Besides GLP-1R, there are several other GPCRs which play a crucial role in human diseases. Despite the significance of GPCRs as therapeutic targets and the excellent drugability of monoclonal antibodies, the use of anti-GPCR therapeutic antibodies in the clinical setting is limited. Despite many years of research, there is, for example, only one FDA-approved “small molecule compound” specific for CXCR4 (plerixafor) approved [80]. Our approach has the potential to accelerate the preparation of natively folded and immobilized membrane protein targets for various screening methods in order to find and validate binders such as antibodies and peptides or to analyze protein–protein interactions. In particular, it opens up a promising methodology for a pre-screening of novel GLP-1R binders, which could reveal new pharmaceuticals to fight hypoglycemia. As GPCR synthesis was conducted in a cell-free system, the introduced technology is well suited for parallelization, automation and high-throughput applications which is of particular interest for drug discovery screening approaches [81,82].

## 4. Materials and Methods

### 4.1. DNA Template Generation

Plasmids for the cell-free synthesis of GLP-1R in eukaryotic lysates were designed according to Brödel et al. [18]. The coding sequence of human GLP-1R (UniProt accession: P43220) was de novo synthesized and cloned in a pUC57-1.8k vector backbone by Biocat GmbH (Heidelberg, Germany). The native signal sequence of GLP-1R was replaced by the signal sequence of honeybee melittin (MKFLVNVALVFMVVYISYIYAD) as it was shown before that the melittin signal sequence leads to an efficient translocation into ER-derived microsomes of *Sf*21 cell lysates, thus enabling subsequent posttranslational modifications such as glycosylation [21]. For purification, a FLAG-tag (DYKDDDDK) and a Strep-tag II© (WSHPQFEK; IBA) were added to the C-terminus of GLP-1R. Besides wildtype GLP-1R, two more GLP-1R sequences were designed and cloned into the pUC57-1.8k vector backbone, one containing an amber stop codon at position L-260 (GLP-1R-L260amb) and another one which included the same mutation but was C-terminally fused to eYFP (GLP-1R-L260amb-eYFP).

### 4.2. Cell-Free Synthesis in Eukaryotic Lysates and Introduction of Non-Canonical Amino Acids into GLP-1R

Within this study, all cell-free reactions were performed in coupled batch mode where translation and transcription are carried out simultaneously. Translationally active lysates derived from *Sf*21 and CHO cells were prepared as described previously [83,84]. A standard batch reaction mixture was composed of 40% (*v*/*v*) cell lysate, a translation mix containing HEPES-KOH (pH 7.6, f.c. 30 mM; BioMol GmbH, Hamburg, Germany), KOAc (f.c. 135 mM, Merck KGaA, Darmstadt, Germany), spermidine (f.c. 0.25 mM, Sigma-Aldrich, St. Louis, MO, USA), Mg(OAc)2 (f.c. 3.9 mM, Merck KGaA) and amino acids (complete 100 µM, Merck KGaA) as well as an energy mix containing creatine phosphate (20 mM, Roche, Grenzach, Germany) ATP (f.c. 1.75 mM, biotechrabbit GmbH, Berlin, Germany), CTP (f.c. 0.3 mM, biotechrabbit GmbH), GTP (f.c. 0.3 mM, biotechrabbit GmbH), UTP (f.c. 0.3 mM, biotechrabbit GmbH) and 0.1 mM (f.c.) m7G(ppp)G (Prof. Edward Darzynkiewicz, Warsaw University, Poland). Additionally, the reaction was supplemented with PolyG primer (IBA, Göttingen, Germany) at a final concentration of 4.5 µM to increase protein yields within the cell-free system as described previously [85]. In order to enable transcription, plasmid DNA (f.c 60 ng/µL) and 1 U/µL T7 RNA polymerase (f.c., Agilent technologies, Waldbronn, Germany) were added. Just as normal batch reactions, so-called no template control (NTC) reactions were prepared as a negative control, in which the DNA template was replaced by ultrapure water. For qualitative and quantitative analysis of cell-free synthesized proteins, 14C-labeled leucine (f.c. 30 µM, specific radioactivity 46.15 dpm/pmol; Perkin Elmer, Baesweiler, Germany) was added to the reaction. For the synthesis of GLP-1R amber mutants cell-free reactions were supplemented with 2 µM (f.c.) Biotin-Lys-tRNA_CUA_ (biotechrabbit GmbH). For the analysis using confocal laser scanning microscopy (CLSM) 2 µM (f.c.), Ala-tRNA_CUA_ (biotechrabbit) was used. If not stated otherwise, cell-free reactions based on *Sf*21 lysate were incubated for 3 h at 27 °C with gentle shaking at 600 RPM in a thermomixer (comfort, Eppendorf, Hamburg, Germany). In contrast, coupled cell-free reactions based on CHO cell lysate were performed at 30 °C for 3 h and 600 RPM. In order to analyze the effect of microsomes on protein translocation and subsequent posttranslational modifications, *Sf*21 and CHO microsome-depleted lysates were generated and used for the cell-free synthesis of GLP-1R. To deplete the microsomes, aliquots of the *Sf*21 and CHO lysate were centrifuged at 16,000× *g* for 10 min at 4 °C. After the centrifugation step, the supernatant of each lysate was collected, while pelleted microsomes were discarded. The microsome-depleted lysate was used as described above (40% (*v*/*v*) cell lysate) for the cell-free synthesis of GLP-1R. In order to enrich the concentration of GLP-1R in the membrane of ER microsomes, a repetitive protein synthesis reaction was performed [86]. GLP-1R was synthesized in four subsequently performed cell-free reactions using the same batch of microsomes. After each synthesis step, microsomes were harvested by centrifugation and applied to the next synthesis reaction resulting in a continuous increase in the GPCR inside the microsomal membranes. Enrichment of GLP-1R was monitored by quantification of the target protein via 14C-leucine labeling.

To investigate the translocation of cell-free synthesized proteins the translation mixture (TM) was separated into the cytosolic (supernatant, SN) and a vesicular fraction (microsomal fraction, MF) by centrifugation at 16,000× *g*, for 10 min at 4 °C. The obtained MF was resuspended in PBS (f.c. 1×, VWR) for further analysis.

### 4.3. Quantitative and Qualitative Analysis of 14C-leucine-Labeled Proteins

The yields of cell-free synthesized proteins were estimated by scintillation measurement due to the statistical incorporation of 14C-leucine in de novo synthesized proteins. For this, an aliquot of 3 µL or 5 µL of the desired synthesis fraction was mixed with 3 mL of a 10% (*v*/*v*) TCA—2% (*v*/*v*) casein hydrolysate (Carl Roth GmbH, Karlsruhe, Germany) solution and boiled for 15 min at 80 °C. After 30 min incubation on ice, samples were transferred to membrane filters (VWR, Darmstadt, Germany) using a vacuum filtration system (Hoefer, Holliston, MA, USA). The filters, which were washed twice with 5% (*v*/*v*) TCA solution and dried with acetone (Carl Roth GmbH), were transferred into scintillation vials (VWR), followed by their resuspension in 3 mL of scintillation cocktail (Quicksafe A, Zinsser analytic, Eschborn, Germany) under agitation for at least 1 h. Scintillation vials were placed into a Hidex SL600 scintillation counter which measured the scintillation signal. The total protein concentration was calculated using the measured scintillation counts, molecular mass and number of leucines specific to the protein of interest and the specific radioactivity. The individual standard deviations calculated for the protein yield were represented by error bars.

For qualitative analysis, cell-free synthesized proteins were separated by SDS-PAGE and subsequent autoradiography. A 5 µL aliquot of the fraction of interest (TM, SN, MF) was incubated with 45 µL MilliQ and 150 µL ice-cold acetone for at least 15 min on ice. Afterwards, precipitated proteins were separated by centrifugation (10 min, 4 °C, 16,000× *g*), the supernatant was removed completely, and the protein pellet was dried for at least 30 min at 45 °C and 1.000 RPM. The dried pellet was resolved in 20 µL 1× LDS sample buffer (NuPAGE™ LDS sample buffer (f.c. 1×); life technologies, Carlsbad, CA, USA) supplemented with 50 mM (f.c.) DTT (Roche) and 8 M (f.c.) Urea (Serva Electrophoresis GmbH, Heidelberg, Germany). Samples were separated on 10% Bis-Tris precast NuPAGE™ SDS gels (Invitrogen, Waltham, MA, USA) for 35 min at 185 V. Subsequently, gels were stained with Coomassie blue solution (SimplyBlue SafeStain, life technologies), transferred on a Whatman paper and dried for 70 min at 70 °C (Unigeldryer, 3545D, Uniequip Laborgerätebau—und Vertriebs GmbH. Planegg, Germany). The dried gel was incubated on a phosphor screen (GE healthcare, Munich, Germany) for at least 3 days and 14C-leucine-labeled protein bands were visualized with Typhoon TRIO+ Imager (GE healthcare).

### 4.4. Silver Staining

Cell-free synthesized proteins were separated by SDS-PAGE and stained with Silver Stain Kit ProteoSilver© Plus (Sigma-Aldrich) according to the manufacturer’s instructions.

### 4.5. Glycosylation Analysis

To verify the glycosylation of cell-free synthesized proteins, an aliquot of the microsomal fraction (MF) was subjected to PNGase F (New England Biolabs, Ipswich, MA, USA) and Endo H (New England Biolabs) digestion. Deglycosylation assays were performed according to the manufacturer’s instructions. A 5 µL aliquot of MF was treated with either PNGase F or Endo H, followed by SDS-PAGE and autoradiography as described above.

### 4.6. Protein Purification and Immobilization on Magnetic Beads

The purification and immobilization of cell-free synthesized proteins in MF was performed using two different types of magnetic beads, SpeedBead Sera-Mag Neutravidin (GE healthcare) and SpeedBead Sera-Mag Streptavidin-blocked Magnetic Particles (GE healthcare). As described above, the protein yield in the MF was determined using hot TCA precipitation to quantify the target protein concentration in each purification strand. The volume of MF, which contained the desired amount of target protein, was filled up to 200 µL with binding buffer containing PBS (f.c. 1×; VWR) with 0.1 % (*v*/*v*) Tween20 (Sigma-Aldrich) and 1% BSA (VWR). The NTC was carried along as a negative control. Thus, it was applied in an equivalent volume as the MF and the same purification protocol was performed for both samples to ensure comparability. The bead suspension was vortexed and the desired volume of beads was transferred to reaction tubes. Afterwards, the beads were washed 3 times in 200 µL of PBS with 0.1 % (*v*/*v*) Tween20 (washing buffer) and blocked for at least 30 min at 4 °C with PBS containing 0.1 % (*v*/*v*) Tween20 and 2% BSA (blocking buffer) under rotation. In order to exchange the buffers, the magnetic beads were collected on the reaction tube wall using a magnetic separator (Promega, Madison, WI, USA). After blocking, beads were washed again 3 times and samples, either cell-free synthesized protein in MF or NTC MF mixed with binding buffer, were incubated with the beads overnight at 4 °C under rotation. Binding supernatant (BSN) was collected for further analysis and beads were washed five times with 200 µL washing buffer. Supernatants of all washing steps were collected and beads were boiled for 10 min at 95 °C in 1 × LDS sample buffer (NuPAGE™ LDS sample buffer supplemented with 50 mM DDT and 8 M Urea) for elution of bound proteins (E1). Then, the collected fractions were further analyzed using TCA precipitation, SDS-PAGE and autoradiography. Scintillation signals were measured as described above.

### 4.7. Analysis of Protein Translocation Using Confocal Laser Scanning Microscopy

The fluorescence of cell-free synthesized GLP-1R amber mutant fused to eYFP was analyzed on µ-ibidi Slides (µ-Slides 18-well, flat, ibidi GmbH, Gräfelfing, Germany) using confocal laser scanning microscopy (CLSM, LSM 510, Carl Zeiss AG, Jena, Germany). A 20 µL aliquot of TM was transferred to a µ-Ibidi-Slide and fluorescence was excited with an argon laser at 488 nm. Brightfield images of the samples were obtained with a helium neon laser at 633 nm. The emission was detected with a band pass filter in the range of 505–550 nm.

For the analysis of the immobilization of GLP-1R-L260amb-eYFP to magnetic beads, the MF was obtained by centrifugation of the translation mixture as described above. SpeedBead Sera-Mag Neutravidin (GE healthcare) beads were washed in 200 µL washing buffer, blocked for 1.5 h at 4 °C in 500 µL blocking buffer and then washed again. Subsequently the beads and the MF were incubated together in washing buffer for 1 h at 4 °C under agitation. Beads were washed twice and an aliquot of 20 µL was transferred to µ-ibidi Slides and analyzed using CLSM as described above.

### 4.8. Bead-Based ELISA

A bead-based ELISA was performed to analyze the accessibility of the ECD of cell-free synthesized microsome-embedded and bead-captured GLP-1R using two anti-GLP-1R-specific antibodies. Beads incubated with either NTC MF or just buffer (=untreated beads) were analyzed as internal controls. The purification of protein-loaded beads was carried out as described above, but instead of eluting the bound protein, the beads were resuspended in washing buffer. An aliquot of 10 µL was obtained for the quantification of bead-captured GLP-1R. The washing buffer was removed and beads were incubated with 200 µL of primary antibody solution for 2 h at 4 °C under rotation. Therefore, monoclonal anti-hGLP-1R (B11) antibody, raised against amino acids 91–145 of GLP-1R (SC-390773-IgG, Santa-Cruz Biotechnology, Dallas, TX, USA) was diluted 1:2000 in binding buffer and human anti-GLP-1R recombinant antibody (clone 3F52, HPAB-0189CQ, creative biolabs, Shirley, NY, USA) was diluted 1:500 in binding buffer. Beads were washed five times using washing buffer before they were incubated for 2 h at 4 °C with 1:3000 (B-11) or 1:1000 (3F52) secondary antibody solution (Goat-anti-mouse IgG (H+L), HRP conjugate (R-05071-500, Advansta Inc, San Jose, CA, USA). After five more washing steps, the beads were incubated with 100 µL TMB (3,3′,5,5′-Tetramethylbezidine, life technologies) substrate solution until a clear blue coloring could be detected. The beads were collected with the magnetic separator and the TMB solution was transferred to a microtiter plate (Corning, Inc., Corning, NY, USA) where the reaction was stopped with 100 µL 0.5 M sulfuric acid. Subsequently, the absorption of the samples was measured by Multi-Mode Microplate Reader FLUOstar Omega (BMG LABTECH, Ortenberg, Germany) at 450 nm and 620 nm as reference. For evaluation, values were subtracted from each other (450–620 nm).

### 4.9. Ligand-Binding Assays

MF containing microsome-embedded GLP-1R was harvested from a standard *Sf*21 in vitro transcription/translation reaction by centrifugation at 16,000× *g* for 10 min and 4 °C. After resuspension in PBS (VWR) GLP-1R MF preparations were incubated for 30 min at 4 °C with SpeedBead Sera-Mag Neutravidin beads (65 µL GLP-1R MF suspension per 100 µg beads, filled up to 500 µL with PBS, containing 0.1% Tween-20) to allow the formation of GLP-1R MF-loaded beads. The concentration of cell-free synthesized GLP-1R in the MF preparation was determined by transcription/translation reaction that was executed in parallel under identical conditions in presence of 14C-leucine to allow radioactive labeling and scintillation measurement of the produced receptor. GLP-1R MF-loaded beads were subsequently washed (1×) and blocked by incubation (for 30 min at 4 °C) with blocking/binding buffer (PBS containing 1 mg/mL BSA (#AM2616, Ambion, Berlin, Germany), 3 mg/mL yeast RNA (Sigma-Aldrich, RR6625) and 0.1 mg/mL sheared salmon sperm DNA (#AM9680, Ambion,). After blocking, GLP-1R MF-loaded beads were incubated for 1 h at 4 °C with the radioactive-labeled exendin-4 peptide ligand (2 nM in 50 µL of blocking/binding buffer), subsequently washed 3 times with PBS containing 0.1% Tween-20, mixed with 2 mL of scintillation cocktail (ROTISZINT^®^ ECO PLUS, Carl Roth GmbH) and transferred to liquid scintillation counting (Wallac 1409 Liquid Scintillation Counter) to determine the percentage of binding exendin-4 ligand. The exendin-4 peptide ligand was generated as radioactive 35S-methionine labeled peptide-mRNA fusion by an in vitro transcription/translation process using a DNA template containing a T7 RNA polymerase promotor, a TMV translation enhancer sequence, an expression cassette for exendin-4, a C-terminal Hisx6 tag coding sequence and a 3′-terminal extension allowing puromycin attachment on mRNA level after in vitro transcription as described in Valencia et al. 2013 [87]. The unspecific, GLP-1R-independent background binding of the exendin-4 ligand to MF-loaded beads was controlled by bead preparations which were loaded either with MF harvested from a NTC reaction or MF containing microsome-embedded GIPR as a control for a non-GLP-1R-related GPCR target. Binding signals of the exendin-4 ligand were calculated as percentage in relation to the respectively used ligand input. Data were obtained from four independent binding experiments with GLP-1R MF and NTC MF-loaded beads, and three independent binding experiments with GIPR MF-loaded beads.

## Data Availability

The data presented in this study are available on request from the corresponding author.

## Supplementary Materials

The following supporting information can be downloaded at: https://www.mdpi.com/article/10.3390/ijms24032808/s1.
