# Supplementary figures and images for: Rapid One-Step Capturing of Native, Cell-Free Synthesized and Membrane-Embedded GLP-1R
Source: Int J Mol Sci. 2023 Feb 1;24(3):2808. doi: 10.3390/ijms24032808 (PMC9917595; doi:10.3390/ijms24032808)

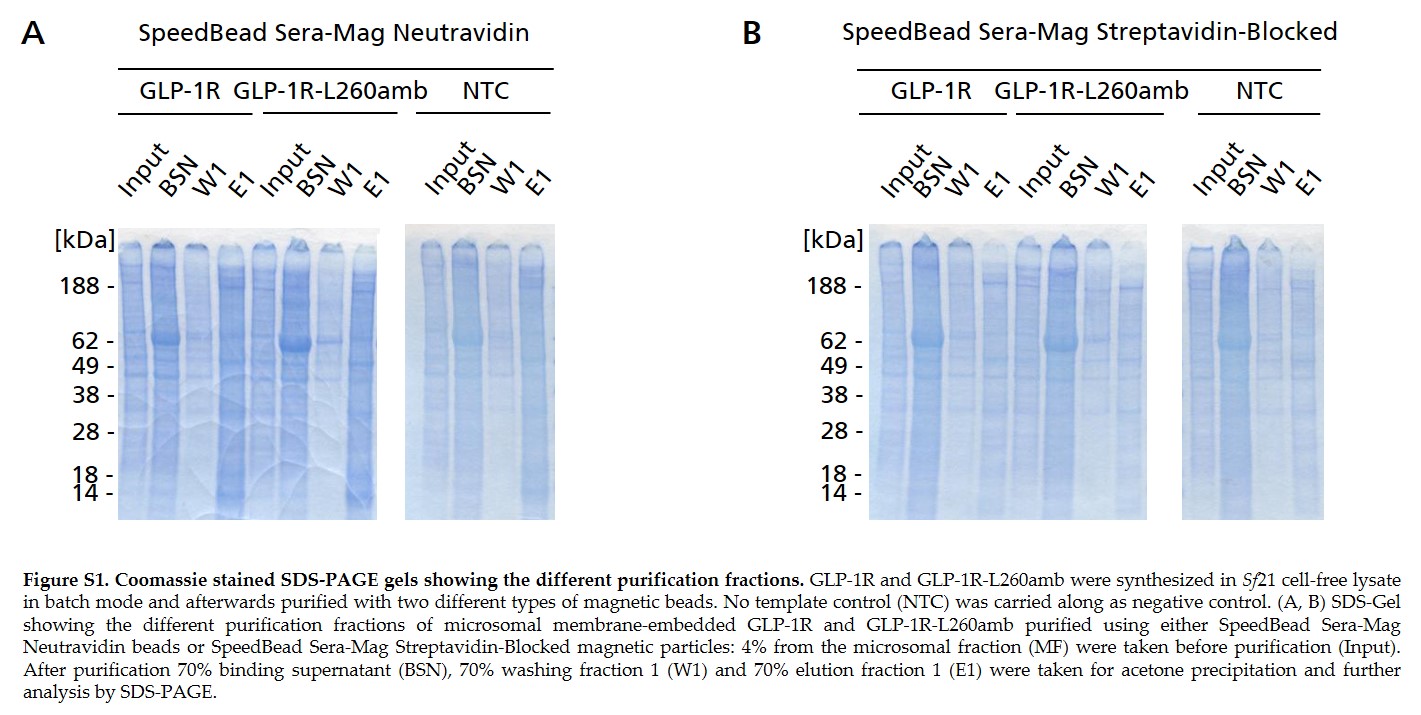

Supplement: Supplementary file 1 [file ijms-24-02808-s001.zip › figure S1.jpg]

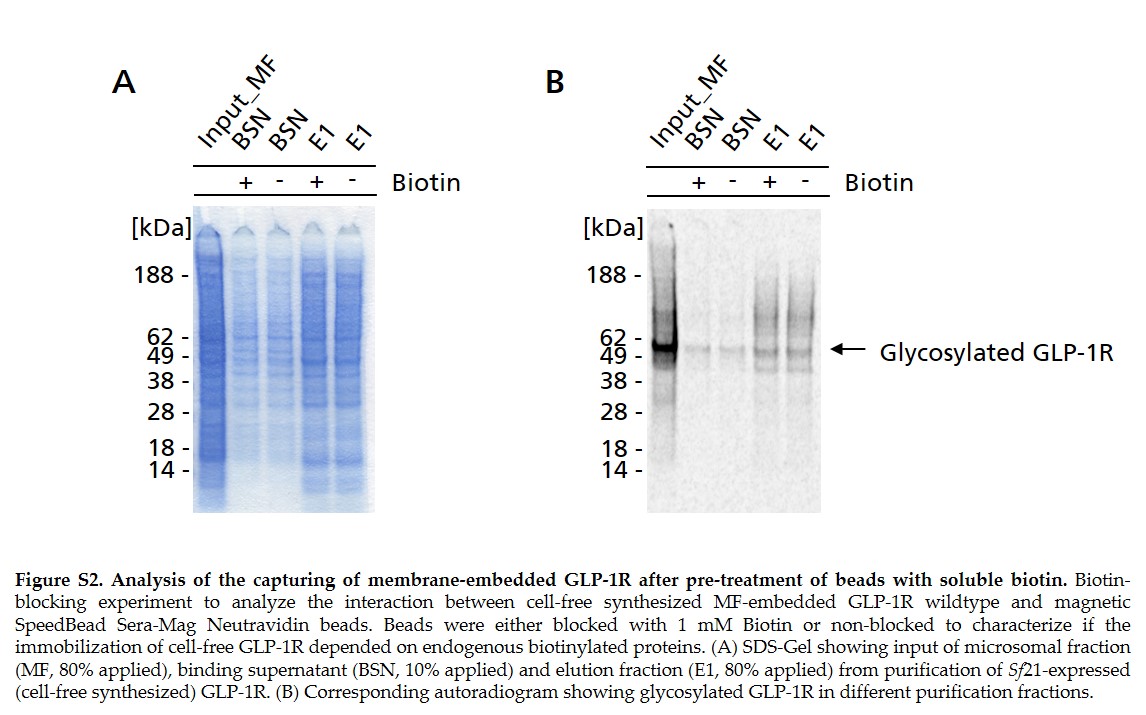

Supplement: Supplementary file 1 [file ijms-24-02808-s001.zip › figure S2.jpg]

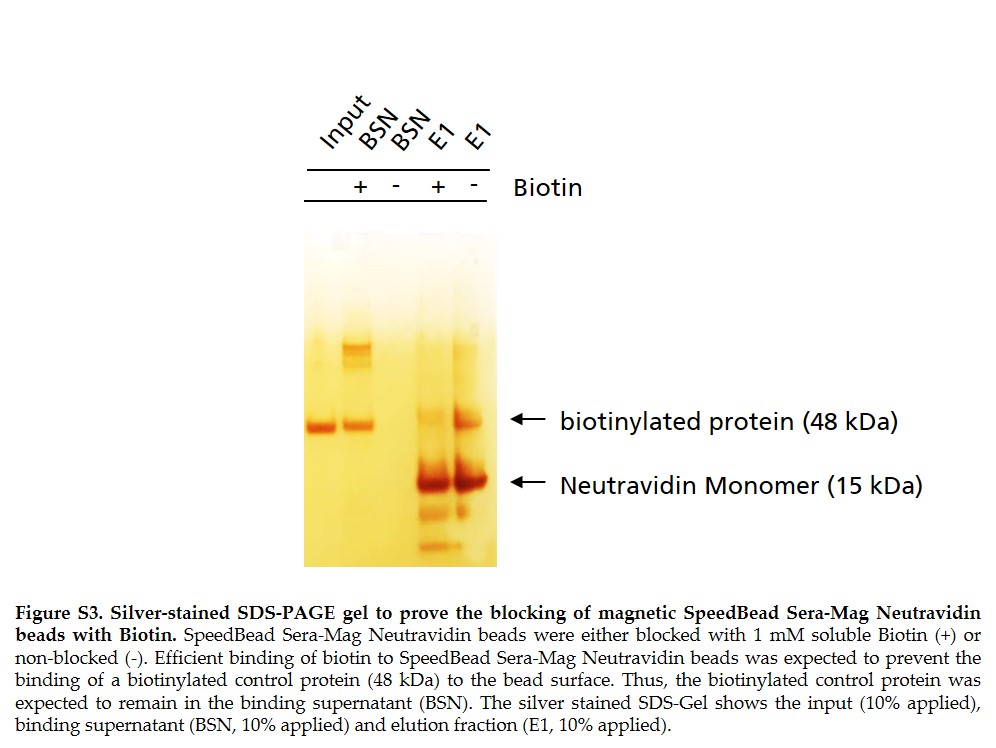

Supplement: Supplementary file 1 [file ijms-24-02808-s001.zip › figure S3.jpg]
